# Congenital megalocornea with zonular weakness and childhood lens-related secondary glaucoma - a distinct phenotype caused by recessive *LTBP2* mutations

**Published:** 2011-10-04

**Authors:** Arif O. Khan, Mohammed A. Aldahmesh, Fowzan S. Alkuraya

**Affiliations:** 1 Division of Pediatric Ophthalmology, King Khaled Eye Specialist Hospital, Riyadh, Saudi Arabia; 2 Department of Genetics, King Faisal Specialist Hospital and Research Center, Riyadh, Saudi Arabia; 3 Department of Pediatrics, King Khalid University Hospital and College of Medicine, King Saud University, Riyadh, Saudi Arabia; 4 Department of Anatomy and Cell Biology, College of Medicine, Alfaisal University, Riyadh, Saudi Arabia

## Abstract

**Purpose:**

To clinically and genetically characterize a distinct phenotype of congenital megalocornea (horizontal corneal diameter ≥13 mm) with secondary glaucoma from spherophakia and/or ectopia lentis during childhood in affected Saudi families.

**Methods:**

Clinical exam, homozygosity scan, and candidate gene analysis.

**Results:**

From 2005 to 2010, eight affected individuals from three consanguineous families were identified. In addition to congenital megalocornea, affected children presented with secondary glaucoma from spherophakia and/or ectopia lentis. One member from each family developed spontaneous complete crystalline lens dislocation into the anterior chamber with associated acute glaucoma during early childhood. Older individuals had phenotypes that would have suggested prior uncontrolled primary congenital/infantile glaucoma had past ophthalmic and/or family histories not been available. Homozygosity mapping performed for the first two families suggested the candidate gene latent transforming growth factor-beta-binding protein 2 (*LTBP2)*, which when sequenced revealed a novel homozgyous mutation that segregated with the phenotype in each family (p.S338P*fs*X4 [c.1012delT], p.Q1619X[(c.4855C>T]). *LTBP2* sequencing in the third family revealed a third novel homozygous mutation (p.C1438Y [c.4313G>A]).

**Conclusions:**

Congenital megalocornea with childhood secondary glaucoma from spherophakia and/or ectopia lentis is a distinct condition caused by recessive *LTBP2* mutations that needs to be distinguished from buphthalmos secondary to primary congenital/infantile glaucoma because typical initial surgical treatment is lens removal in the former and angle surgery in the latter. Complete dislocation of the crystalline lens into the anterior chamber during early childhood can occur in young children with this unique phenotype.

## Introduction

Primary congenital/infantile glaucoma is an isolated developmental abnormality of the anterior chamber drainage angle for which the most common identifiable cause is recessive cytochrome P450 subfamily 1 polypeptide 1 (*CYP1B1*) mutations [1,2]. Resultant increased intraocular pressure (IOP) within the first few years of life causes the classic signs of primary congenital/infantile glaucoma: buphthalmos, corneal haze/scarring, Descemet membrane breaks (Haab striae), optic nerve cupping, and myopia with astigmatism. In severely buphthalmic eyes, ectopia lentis and retinal detachment can occur [3]. Surgical management of primary congenital/infantile glaucoma often begins with angle surgery and can include goniotomy, trabeculotomy, trabeculectomy, and glaucoma implant devices [4].

Although the classic phenotypic features of primary congenital/infantile glaucoma typically allow for a straightforward diagnosis, similar signs can be part of other pediatric conditions that are sometimes mistaken as early childhood glaucoma [5]. Over the last several years we have become aware of a distinct familial ocular syndrome of congenital megalocornea with childhood-onset secondary glaucoma from spherophakia and/or ectopia lentis that can resemble the buphthalmos of primary congenital/infantile glaucoma, particularly in older affected individuals with advanced glaucomatous damage. A review of the literature reveals that this ocular syndrome has been described in three consanguineous families (Turkish, Moroccan, and Macedonian), two of which recently underwent genetic analysis and were found to harbor mutations in latent transforming growth factor-beta-binding protein 2 (*LTBP2*) [6,7]. The purpose of our report was to characterize three Saudi families that we have diagnosed with this unique phenotype both clinically and genetically.

## Methods

Institutional review board approval was obtained for this study. Three consecutive families (eight patients) who were referred to one of the authors (A.O.K.) from 2005 to 2010 and were recognized to have a distinct phenotype of congenital megalocornea with childhood-onset secondary glaucoma from spherophakia and/or ectopia lentis were prospectively enrolled in the study. Patients underwent complete ophthalmic examination, pediatric evaluation, and ophthalmic treatment as necessary. DNA was extracted from whole blood samples of patients. The first two identified families were consanguineous and thus homozygosity mapping was performed to investigate an autosomal recessive cause for the phenotype using previously-described methodology [8]. Briefly, genomewide single nucleotide polymorphism (SNP) genotyping was performed using Affy 250K StyI SNP Chip (Affymetrix, Inc., Santa Clara, CA) as per the manufacturer's instructions; homozygosity mapping and cataloging of genomic regions were performed with the software AutoSNPa software (University of Leeds, Leeds, UK). This strategy allowed selection of candidate genes for direct sequencing. The most likely candidate gene was sequenced for all three enrolled families.

For DNA sequencing of the identified candidate gene, polymerase chain reaction (PCR) amplification was performed on a thermocycler (DNA Engine Tetrad; MJResearch, Inc., Hercules, CA) in a total volume of 25 µl, containing 10 ng DNA, 50 mM KCl, 10 mM Tris-HCl (pH 9.0), 1.5 mM MgCl_2_, 0.1% Triton X-100, 0.25 mM of each dNTP, 0.8 µM of each primer and 0.5 units of Taq polymerase (D-40724; QIAGEN, Hilden, Germany). For PCR, an initial denaturation step at 95 °C for 10 min was followed by 40 cycles of denaturation at 95 °C for 30 s, annealing at 59 °C for 30 s, and extension at 72 °C for 30 s, followed by a final extension step of 72 °C for 10 min. All exons and intronic boundaries of the selected candidate gene were sequenced using an Amersham ET Dye Terminator Cycle Sequencing Kit (Amersham Biosciences, Piscataway, NJ) following the manufacturer’s instructions. Sequence analysis was performed using the SeqManII module of the Lasergene (DNA Star Inc. Madison, WI) software package using reference DNA sequences from GenBank for comparison. 

## Results

### Clinical

**Figure 1:**
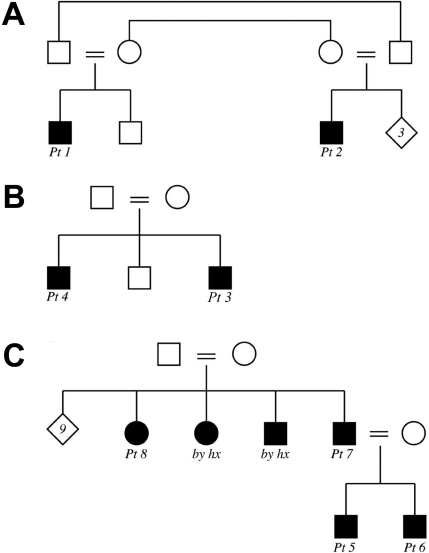
Pedigrees of the families. The pedigrees for Family 1 (**A**), Family 2 (**B**), and Family 3 (**C**) are shown. Pt: patient; by hx: affected by history but not available for the study.

The clinical histories of the families, summarized in Figure 1 and Table 1, are further detailed below.

**Table 1:** Clinical summary.

**Patient**	**Family**	**Sex**	**Original referral**	**Original referral findings**	**Later course**
1	1	M	2y	megalocornea, lens subluxation	acute lens-related pupillary block glaucoma several months after referral
2	1	M	1y	megalocornea, lens subluxation	glaucoma surgery followed by lens luxation, retinal detachment, and phthisis
3	2	M	4m	nystagmus, megalocornea, spherophakia	lens subluxation (4y), acute lens-related pupillary block glaucoma (6y)
4	2	M	4y	megalocornea, spherophakia, lens-related pupillary block glaucoma	stable after lensectomy and anterior vitrectomy
5	3	M	1.5y	megalocornea, lens subluxation, acute lens-related pupillary block glaucoma	stable after lensectomy and anterior vitrectomy
6	3	M	6m	megalocorrnea, spherophakia	acute lens-related pupillary block glaucoma 1year after referral
7	3	M	2y	records not available – multiple surgeries	phthisis
8	3	F	10y	megalocornea, lens subluxation	lenses eventually luxated posteriorly and surgery not done, has glaucoma at age 20y

M: male; F: female; Original referral: age at time of original referral; y: years old; m: months old.

### Family 1: (two affected cousins, Figure 1A)

#### Patient 1

A two-year-old boy was referred for glaucoma surgery. He had a history of large corneas since birth and a family history for congenital glaucoma in his cousin. Birth history was unremarkable and the child was otherwise normal without dysmorphic features or habitus. The parents were first cousins and two older siblings were normal by history. The child could fixate well with both the right eye (OD) and left eye (OS) and there was no strabismus. There was occasional fine pendular low-amplitude high-frequency nystagmus. Ophthalmic examination was significant for clear corneas with 13 mm horizontal corneal diameter (without breaks or scarring), bilateral 360° mild ectropion uveae, and bilateral lens subluxation within the pupillary margin (inferotemporally OD and inferonasally OS). By the Tonopen (Reichert, Inc., Depew, NY), while the child was sleeping, the IOP was 18 mmHg in both eyes. Fundus examination was unremarkable, with healthy optic nerve heads without cupping. Retinoscopy through the aphakic portion of the pupil was +11 diopters (D) OD and +12 D OS. Six months later the patient presented with acute irritability and vomiting and red eye OS. Examination was significant OS for conjunctival injection, corneal edema, shallow anterior chamber, and complete crystalline lens dislocation into the anterior chamber causing pupillary-block glaucoma. Examination OD revealed anterior tenting of the iris by the subluxated crystalline lens. Tonopen IOP was 30 mmHg OD and over 50 mmHg OS. The child underwent bilateral lensectomy and anterior vitrectomy. Homocystinuria screening (urine cyanide nitroprusside test) was negative. Post-operatively he was fitted with aphakic glasses and over a three-year post-operative period Tonopen IOP has remained <20 mmHg in both eyes without evidence for glaucoma.

#### Patient 2

**Figure 2:**
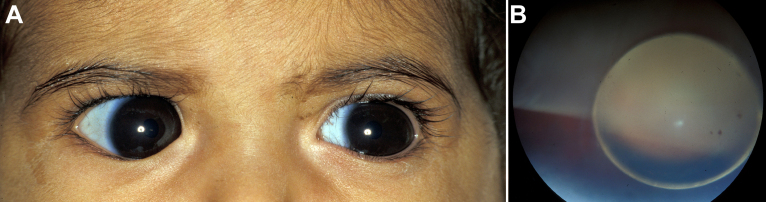
Family 1: cousin of the proband. **A**: At one year of age, large corneas are evident (14 mm horizontal diameters) but without corneal haze/scarring or Descemet breaks. **B**: After primary Ahmed valve implantation in both eyes, the crystalline lens dislocated into the posterior chamber and retinal tears developed bilaterally. The right eye is shown.

The proband's cousin with a history of congenital glaucoma was recalled and examined. He was a 14-year-old boy with pre-phthisical eyes and a history of multiple intraocular surgeries. His parents were cousins. He was tall and thin with a relatively high arched palate, but had no other features to suggest Marfan syndrome (no arachnodactyly, no chest depression, no relatively increased arm span, no electrocardiographic abnormalities). A review of the records revealed that at one year of age he underwent his first ocular surgery, bilateral primary Ahmed valve implantation for presumed primary congenital/infantile glaucoma. However, although megalocornea (horizontal corneal diameters of 14 mm) was documented preoperatively, other expected phenotypic features for buphthalmos from primary congenital/infantile glaucoma were not (e.g., corneal haze/scarring, Descemet breaks, optic disc cupping; Figure 2A). Despite uncomplicated surgery, post-operatively the child developed complete posterior dislocation of the crystalline lenses into the posterior vitreous cavities with giant retinal tears bilaterally (Figure 2B).

### Family 2: (two affected brothers, Figure 1B)

#### Patient 3

**Figure 3:**
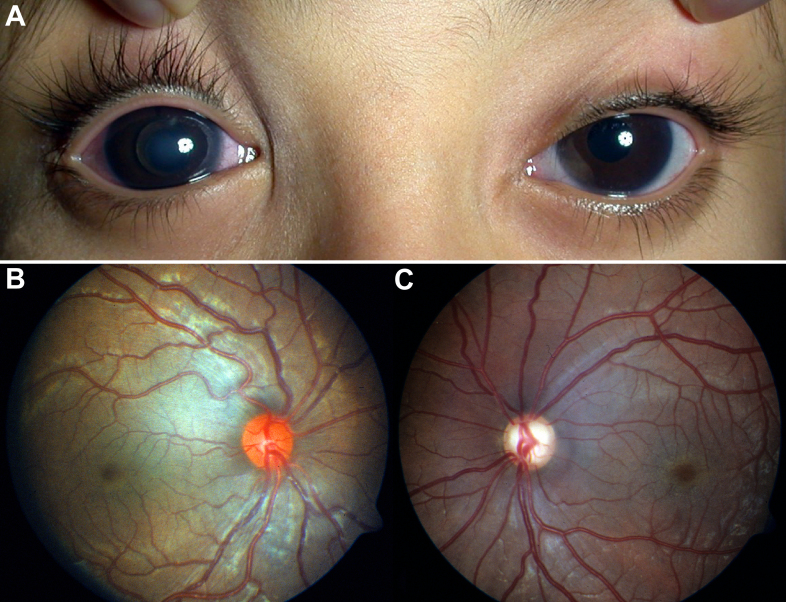
Family 2: two affected brothers. **A**: At six years of age, the proband developed right acute pupillary block glaucoma. Complete crystalline lens dislocation into the anterior chamber of the right eye can be seen. In the left eye, inferotemporal crystalline lens subluxation can be appreciated. Both corneas are symmetrically enlarged (14 mm horizontal diameter). **B**, **C**: The proband's brother had lens-related pupillary block glaucoma in the left eye for which he had bilateral lensectomy and anterior vitrectomy at four years of age. At ten years of age, glaucomatous cupping in the left eye (**C**) as opposed to the right eye (**B**) can be appreciated. Megalocornea (not shown) was symmetric (14 mm horizontal corneal diameters without scars or breaks).

A four-month old boy was referred for congenital glaucoma surgery. He had a history of large corneas since birth and a family history for childhood glaucoma in one of his two older brothers. Birth history was unremarkable and the child was otherwise normal without dysmorphic features or habitus. Parents were first cousins. The child could fixate well with either eye and there was no strabismus. There was occasional fine pendular low-amplitude high-frequency nystagmus. Ophthalmic examination was significant for megalocornea (14 mm horizontal diameter without breaks or scarring), bilateral iridonesis, and bilateral forward iris tenting by the lens (bilateral spherophakia). By the Tonopen IOP while the child was sleeping was 22 mmHg OD and 23 mmHg OS. Fundus examination was unremarkable; optic nerve heads were healthy without cupping. Retinoscopy was −22 D in both eyes and was prescribed as glasses. Homocystinuria urine screening was negative. At four years of age the child had bilateral lens subluxation within the pupil (inferotemporally OD and inferonasally OS) for which the refraction through the aphakic portion of the pupil of +16 D in both eyes. By the Tonopen IOP was 16 mmHg in both eyes. At six years of age the child presented with eye pain and redness OD, which the father complained was increasingly occurring on and off over the previous year. There was complete crystalline lens dislocation into the anterior chamber OD causing pupillary-block glaucoma (Figure 3A). The ectopic left crystalline lens was tenting the iris anteriorly. By the Tonopen IOP was 50 mmHg OD and 26 mmHg OS. Bilateral lensectomy with anterior vitrectomy was performed. Post-operatively the aphakic refraction was +15 D OD and +18 D OS and vertical cup-to-disc ratio was 0.4 OD and 0.3 OS. The child was fitted with aphakic glasses and was prescribed timolol 0.5% twice daily OD. Tonopen IOP has ranged in the low twenties for both eyes over a four-year post-operative period without evidence for glaucoma.

#### Patient 4

The proband's 13-year-old older brother with a history of congenital glaucoma was recalled and examined. At four years of age he had been diagnosed as megalocornea with spherophakia bilaterally with pupillary-block glaucoma OS, for which he underwent bilateral lensectomy and anterior vitrectomy. At 13 years of age he had bilateral symmetric megalocornea (14 mm horizontal corneal diameters without scarring or breaks) and was otherwise normal without dysmorphic features or habitus. He was taking timolol 0.5% twice daily in both eyes. Applanation tonometry IOP was 20 mmHg in both eyes and there was evidence for previous glaucomatous damage OS (Figure 3B,C). He was aphakic with a refraction of +10 diopters in both eyes (visual acuity with correction 20/20 OD, 20/200 OS) and without evidence for glaucoma OD.

### Family 3: (two affected brothers, affected father, affected paternal aunt; Figure 1C)

#### Patient 5

**Figure 4:**
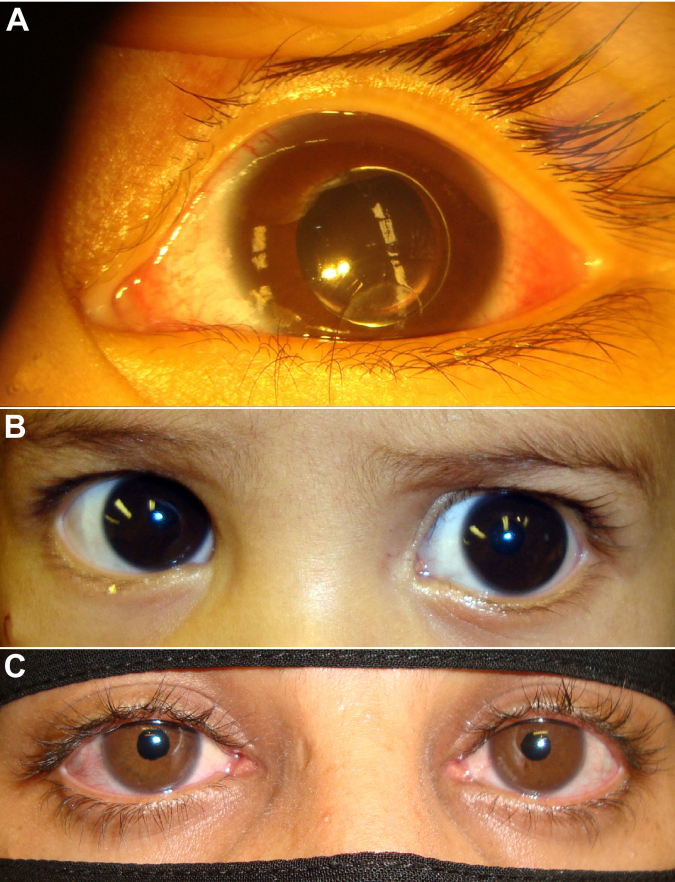
Family 3: two affected brothers, affected paternal aunt. **A**: At one and one-half year of age, the proband developed left acute pupillary block glaucoma. Complete crystalline lens dislocation into the anterior chamber of the left eye and a large corneal diameter (14 mm horizontally) can be seen. **B**: The proband's six-month-old brother was tentatively scheduled for primary congenital glaucoma surgery by his physician. Megalocornea is evident (14 mm horizontal diameter without breaks or scarring). The child also had bilateral spherophakia (not shown). **C**: The paternal aunt of the proband had been diagnosed with glaucoma at ten years of age but never had surgery. At 20 years old, bilateral symmetric megalocornea (14 mm horizontal diameter without breaks or scarring) is evident. Both crystalline lenses were posteriorly dislocated (not shown). She had high intraocular pressure, angle synechiae, and glaucomatous optic nerve damage in her right eye (not shown).

A one and one-half year old boy presented to the emergency department with a three-day history of eye pain OS and irritability. Prior to presentation he had had episodes of left red painful eye on and off over the preceding few months. He had a history of large corneas since birth and a family history for congenital glaucoma in his younger brother, his father, and three of his father's siblings. Birth history was unremarkable and the child was otherwise normal by history. Parents were first cousins. The child could not be examined while awake because of irritability and thus examination was performed under chloral hydrate sedation. He did not have dysmorphic features or habitus. Ophthalmic examination was significant OD for 13 mm horizontal corneal diameter (without breaks or scarring) and inferonasal lens subluxation. Ophthalmic examination OS was significant for conjunctival injection, a 13 mm corneal diameter, corneal edema and central scarring, and complete crystalline lens dislocation into the anterior chamber causing pupillary block (Figure 4A). By the pneumotonometer (Model 30 Classic; Mentor, Norwell, MA) IOP was 15 mmHg OD and 54 mmHg OS. The child underwent emergent bilateral lensectomy and anterior vitrectomy. Three months post-operatively the child was comfortable, Tonopen IOP was 15 mmHg in both eyes, aphakic refraction was +18 D in both eyes, and optic nerve heads were healthy without cupping in both eyes. Homocystinuria screening was negative. For one year post-operatively he has not had evidence for glaucoma.

#### Patient 6

The child's younger six-month-old brother was recalled for examination. He was scheduled to undergo surgery for primary congenital glaucoma by another physician. The child was carefully examined under chloral hydrate sedation. He was not dysmorphic. He had bilateral megalocornea (14 mm horizontal diameter without breaks or scarring; Figure 4B), bilateral iridonesis, and bilateral anterior tenting of the iris by the lens (bilateral spherophakia). By the Tonopen IOP while the child was sleeping was 19 mmHg OD and 20 mmHg OS. Fundus examination was unremarkable, with healthy optic nerve heads without cupping. Retinoscopy was −22 D in both eyes. The other physician was alerted, angle surgery for primary congenital/infantile glaucoma was canceled, and bilateral lensectomy/anterior vitrectomy was recommended but the family refused. Several months later the child presented with a right red painful eye from acute pupillary glaucoma (Tonopen IOP 50 mmHg OD), at which time he underwent bilateral lensectomy and anterior vitrectomy.

#### Patient 7

The father was recalled and examined. By history he had had multiple surgeries for childhood glaucoma. Upon examination he had bilateral phthisis. He was tall and thin with a relatively high arched palate and had mild pectus excavatum, but had no other features to suggest Marfan syndrome (no arachnodactyly, no relatively increased arm span, no electrocardiographic abnormalities) and was otherwise normal without dysmorphic features or body habitus.

#### Patient 8

One of the father's affected siblings (the father's sister) was recalled and examined. She was diagnosed with glaucoma at 10 years of age for which she was prescribed ophthalmic drops but for which she did not undergo surgery. At 20 years of age megalocornea was evident (14 mm horizontal corneal diameter without breaks or scarring; Figure 4C) but otherwise she did not have dysmorphic features or body habitus. In addition to megalocornea, she had bilateral iridonesis, bilateral mild ectropion uveae with an irregular pupillary border, and bilateral complete crystalline lens dislocation into the posterior vitreous. Applanation tonometry was 35 mmHg OD and 16 mmHg OS (using dorzolamide 2% three times a day in both eyes and timolol 0.5% twice a day in both eyes). There was almost complete optic nerve cupping OD and a healthy central 0.3 cup-to-disc ratio OS. Gonioscopy revealed 360 degrees peripheral anterior synechiae OD but not OS. Aphakic refraction was +9.00 diopters in both eyes. She was referred for further glaucoma management.

### Molecular genetics

#### Homozygosity mapping

While several runs of homozygosity (ROH) were identified in the four affected individuals from Families 1 and 2, all four individuals had a common ROH on 14q that contained *LTBP2*. Because *LTBP2* had just been implicated in this ocular syndrome as we had started our analyses [7], it was selected as a candidate gene for sequencing.

#### Candidate gene sequencing

**Figure 5:**
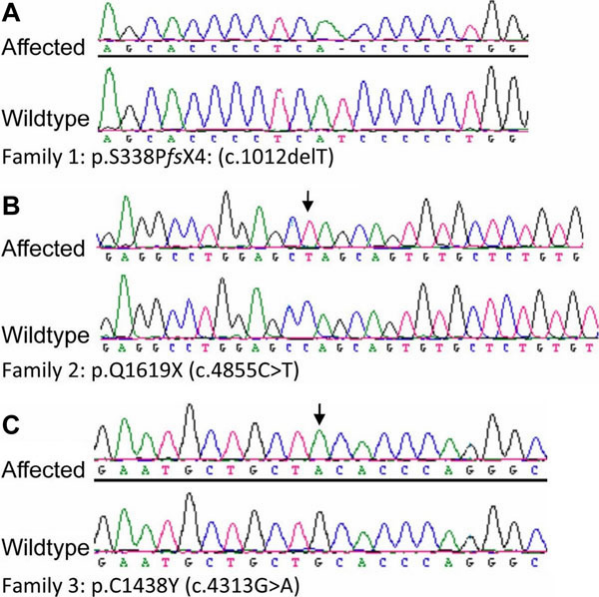
Genetic analysis. **A**, **B**, **C**: Sequencing of *LTPB2* revealed a novel homozygous mutation in each family that segregated with the phenotypes.

*LTBP2* was amplified using primers (Table 2) that cover the entire coding region followed by bidirectional sequencing in all eight examined affected individuals from the three families. Three novel homozygous *LTBP2* mutations were identified in the eight patients, one in each family: c.1012delT (p.S338P*fs*X4) in exon 4 for Family 1, c.4855C>T (p.Q1619X) in exon 33 for Family 2, and c.4313G>A (p.C1438Y) in exon 29 for Family 3 (Figure 5). For Family 1 and Family 2, the unaffected parents were confirmed to be heterozygous for the respective mutations. For Family 3, the affected father and paternal aunt were homozygous for the p.C1438Y mutation while the unaffected mother was a carrier. This missense mutation mutation in *LTBP2* results in loss at position 1438 of cysteine, a nonpolar hydrophobic amino acid that is important in disulfide bonds and protein folding and is conserved among species (Table 3). It was not present in 100 ethnically-matched normal controls.

**Table 2:** *LTBP2* primers.

**Exon ID**	**Forward**	**Reverse**
1a	CCCAGAGCAGGAGAAAGG	GGAACAGACTGTACACCTTGG
1b	GCCCCCTAGACTCAGAGAAG	AATCTTCCAATCCCGATTTT
2	AATGGCAGAGTCAGGATTCA	CTTCAGGACGCAGACTAGGA
3	CTGAGGCCAGGAGAGTGG	CCAGCCCCAACACCTACT
4	AAGCCTGGTGATTCCACATA	CACAAAGCAGGTGCTCAAC
5	GCGTCCAGTAGGTACTCAGC	AGCTAGGCTGCCAAGTGAG
6	GGGGCTGGTTATTATCCACT	GGCTGAGAAGTTGAGGGAAT
7	GGGATCATTCTGGGGTTCTA	CTGTGTGCCTGGTATTGACA
8	ACTCCCTTCTCCCCTTCTTT	ACAGACTGCACCAGCAGAG
9	GCTGAGAGGAGTCTGGTGAG	TGGCTTCCTCTGTCACTCTC
10	GGAGAGGAATCCCACTGAAT	ATCTCTGTTCCAGCAGGATG
11	ATTCCACTACGCCTCTTCCT	GCAGGGAAGGCTACTTCAG
12	ACGTGCTTATCCCAACCTG	TCTTGACCCCATATGGAAGA
13	AAGAGTCCACGCTTTCTGTG	ATGGCTGCTCCATAAACAAG
14	GTAAAGTGCCTGGCAGAATG	GGTGTATAGAGAGCTCCCAGAA
15	TTAGACTGGATGTGCTCCAAC	AGAGGGACCCTGTGTTCTTT
16	CCCCTAGGGTCTTATGCAAG	GAGACTGGTCTTCCCCTGAA
17	CCCACTGGGCTGACTTTAT	AGGCTGGAGTTCTGGTCTCT
18	GGGCCTGAGCTAGATCATTT	AAGGGCTCAGGAATTCTCAT
19	GGCAGCTCTCATTCTTTCCT	TGAATATGGCCAAAGAGGAG
20–21	CATGCAGAGTGCTCTGAGTTAC	GGTCCATTTATGGGGTCTTC
22	TTCTAGGGAGGGGGTTTTAG	AAGCTTGTGAGCGACTCTTG
23–24	CCCAAGAGTCGCTCACAA	ACTCCTCGCTCCCATCTTC
25	CGAGCCTTTTCCTACATAAGC	CAGCACGAAGATGATGATTG
26–27	GGAATAGATCAAGAACCCCAGA	CTTCTTTGAAGCCTCCCTTG
28	TCTGTCCATTGGTTCCTCCT	TGTAGCTCCTGGTTTTGCTG
29–30	GGCCACTTCTTAGGGTTGTG	ACAGAAAAGGTGGAGGCAAC
31	GTAGGAACCGGAGGCAAG	CCTGGGGACAATCTCTGAC
32–33	GTGGGCTGTCAGAGATTGTC	CTACTTTGTCCCCAAACAGC
34	ATCTCCCAGAGGGTACCAGT	CCTGGGCGTATGTACTTGTC
35	TCCACAAGAATTTTATGATCCTC	TTGTCTTTTGTCTGGGAACC
36	TGTCCTTGAGTTGCTTGGTT	TCAGGATGATGGTGGATTGT

**Table 3:** Conservation analysis of Family 3 mutation c.4313G>A).

** **	** **	** **	** **	** **	**NM_000428.2 (LTBP2_i001): p.(Cys1438Try) ↓**	** **	** **	** **	** **
**Species**	**Amino Acid**								
Human	A	G	G	T	C	C	E	A	Q
Mouse	A	G	G	T	C	C	E	A	Q
Dog	A	G	G	T	C	C	E	A	Q
Elephant	A	G	G	T	C	C	E	A	Q
Opossum	A	G	G	T	C	C	E	E	R
Chicken	A	G	V	T	C	C	E	Q	K
X_tropicalis	E	G	I	S	C	C	E	D	R
Zebrafish	Q	G	A	T	C	C	E	Q	L

Data from Multiz Alignment (UCSC Human Genome Browser) showing high level of conservation of C1438 across species.

## Discussion

Eight patients from three consanguineous families had congenital megalocornea and childhood secondary glaucoma from spherophakia and/or ectopia lentis, a condition that can resemble buphthalmos from primary congenital/infantile glaucoma. The older affected patients had end-stage phenotypes that would have been difficult to distinguish from previous uncontrolled primary congenital/juvenile glaucoma had prior family history and/or ophthalmic records not been available, particularly because of the confounding factor of megalocornea. One affected individual from each family developed complete spontaneous crystalline lens dislocation into the anterior chamber and associated acute glaucoma as a young child. For each family, findings segregated with homozygous *LTBP2* mutations.

In 1991 Bjerrum and Kessing [6] suggested the existence of a recessive phenotype of childhood secondary glaucoma from early-onset ectopia lentis causing pupillary block and anterior synechiae in a consanguineous Turkish family. Their conclusions were based on examinations of four affected children examined before two years of age. A fifth affected child was examined at nine years of age at which time she had bilateral phthisis; the authors noted that she would not have been suspected of having this unique diagnosis if the other affected children had not been examined at a young age [6]. Congenital megalocornea was not suspected; the observed large corneal sizes were thought to be related to buphthalmos from the secondary glaucoma. Nineteen years later in 2010 Desir and colleagues [7] described three children from a consanguineous family of Morrocan descent and one child from a consanguineous family of Macedonian descent with congenital megalocornea and childhood secondary glaucoma from spherophakia and/or ectopia lentis. Although the oldest Morrocan child (14 years old) had tall stature and a narrow face suggestive of Marfan syndrome, other Marfan syndrome criteria were not met and fibrillin-1 testing was negative. By performing homozygosity mapping for the Morrocan family, the authors identified *LTBP2* as a candidate gene and subsequent sequencing revealed homozygous *LTBP2* mutations to underlie the phenotype in both the Morrocan family (p.Val600Gly*fs*X2) and the Macedonian family (p.Arg299X) [7]. In the current study we clinically characterize eight patients from three consanguineous Saudi families with congenital megalocornea and childhood secondary glaucoma from spherophakia and/or ectopia lentis and confirm its recurrent association with *LTBP2* mutations.

Several patients in our series were previously diagnosed as primary congenital/infantile glaucoma and thus the unique phenotype of congenital megalocornea with childhood zonular weakness and lens-related secondary glaucoma may be more common than is currently recognized. The combination of large corneas with elevated IOP is very suspicious for primary congenital/infantile glaucoma in a child or in an adult; however, for this unique *LTBP2*-related phenotype the megalocornea is unrelated to glaucoma with the primary problem being spherophakia and/or ectopia lentis. Spherophakia and/or eventual ectopia lentis can cause recurrent pupillary block with intermittent IOP spikes, leading to angle changes and eventual secondary glaucoma. For this mechanism of glaucoma, angle surgery is not appropriate although it is a common first line treatment for primary congenital/infantile glaucoma [4].

One child from each family in this series developed spontaneous complete crystalline lens dislocation into the anterior chamber with acute glaucoma during early childhood, an unusual ophthalmic presentation that has previously been most strongly associated with homocystinuria although not typically at such a young age as in our series (1–6 years of age in our series as opposed to 15 years of age in one study [9]). Complete spontaneous crystalline lens dislocation into the anterior chamber is not typical for other conditions that can include spherophakia and/or ectopia lentis, such as Marfan syndrome, Weill-Marchesani syndrome, ectopia lentis et pupillae, and idiopathic isolated ectopia lentis [9-11]. Although older individuals in our series (Patients 2 and 7) had tall stature and a high arched palate, other individuals had no evidence for dysmorphic features, dysmorphic body habitus, or non-ocular congenital abnormalities.

The potential role of *LTBP2* in ocular development was first highlighted when *LTBP2* mutations were found in four Pakistani families (p.A138P*fs*X278, p.R299X, p.E415R*fs*X596, p.Q111X), eight European Gypsies (p.R299X), and three Iranian families (p.S472fsX3, p.Y1793*fs*X55) who were diagnosed with *CYP1B1*-negative primary congenital/infantile glaucoma [12,13]. However, many of those patients were carefully examined later in life at which time many had corneal scarring that limited ophthalmic examination. Although their phenotypes could be compatible with previously uncontrolled primary congenital/infantile glaucoma, one cannot rule out an actual diagnosis of congenital megalocornea with childhood ectopia lentis and lens-related secondary glaucoma. Consistent with this possibility is the fact that several of those patients with corneas clear enough to allow visualization of the anterior segment were documented to have ectopia lentis [12,13], an uncommon feature of primary congenital/infantile glaucoma. Moreover, patients diagnosed as primary congenital/infantile glaucoma from *LTBP2* mutations had poorer results from angle surgery as children [13,14], which would be expected if the actual diagnosis were congenital megalocornea with childhood secondary lens-related glaucoma.

Another phenotype that has been associated with *LTBP2* mutations is isolated spherophakia and/or ectopia lentis without megalocornea [15]. In one South Indian consanguineous family an underlying recessive frameshift mutation in LTBP2 predicted to replace the terminal six amino acids of the protein with 27 novel amino acids (p.H1816P*fs*X28) was associated with the phenotype, which did not include glaucoma at the time of the study in any of the three affected siblings (10, 18, and 21 years of age) [15].

LTBP2 is a member of the latent transforming growth factor-beta-binding family of proteins that is expressed in the anterior segment of the eye, particularly at the ciliary body [12,13]. Although its exact function is unknown, LTBP2 structurally resembles and interacts with the protein fibrillin-1, a main component of extracellular microfibrils [7,12,13,15]. Mutations in fibrillin-1 cause fibrillinopathies of which the most well known is Marfan syndrome, which sometimes includes megalocornea and usually includes ectopia lentis without complete lens dislocation [11]. A normal functional role of LTBP2 in the fibrillin pathway is presumably why mutations in *LTPB2* cause congenital megalocornea and early childhood ectopia lentis with a tendency for complete lens dislocation. Fibrillin-1 aggregates also normally bind the large latent complex of the cytokine transforming growth factor β (TGF-β); in fibrillinopathies such as Marfan syndrome failure of this event results in increased TGF-β activation and systemic connective tissue manifestations such as aortic aneurysm, heart valve degeneration, and potential pneumothorax [16]. However none of the patients with *LTBP2* mutations reported to date [7,12,13,15], including ours, have evidence for clinically significant systemic manifestations of increased TGF-beta activation. This may be because of the expression pattern of LTBP2 or because of redundancy with other LTBPs. In total eight different homozygous *LTBP2* mutations have been previously reported [7,12,13,15], all of which were predominantly ocular phenotypes. We document phenotype-genotype correlation for an additional three homozygous *LTBP2* mutations. Table 4 lists published *LTBP2* mutations to date.

**Table 4:** *LTBP2* mutations to date.

**#**	**Ethnicity**	**Homozygous mutation**	**Exon**	**Protein effect**	**Described phenotype**	**Reference**
1	Pakistani	c.412delG	1	p.A138PfsX278	diagnosed as primary congenital glaucoma	[13]
2	Pakistani	c.331C>T	1	p.Q111X	diagnosed as primary congenital glaucoma	[13]
3	Pakistani	c.1243_1256del14	6	p.E415RfsX596	diagnosed as primary congenital glaucoma	[13]
4a	Gypsy	c.895C>T	4	p.R299X	diagnosed as primary congenital glaucoma	[13]
4b	Macedonian	c.895C>T	4	p.R299X	primary megalocornea & spherophakia	[7]
5	Iranian	c.1415delC	7	p.S472fsX3	diagnosed as primary congenital glaucoma	[12]
6	Iranian	c.5376delC	36	p.Y1793fsX55	diagnosed as primary congenital glaucoma	[12]
7	Moroccan	c.1796dupC	9	p.V600GfsX2	primary megalocornea & secondary lens-related glaucoma	[7]
8	South Indian	c.5446dupC	36	p.H1816PfsX28	spherophakia	[15]
9	Saudi	c.1012delT	4	p.S338fsX4	primary megalocornea & secondary lens-related glaucoma	current study
10	Saudi	c.4855C>T	33	p.Q1619X	primary megalocornea & secondary lens-related glaucoma	current study
11	Saudi	c.4313G>A	29	p.C1438Y	primary megalocornea & secondary lens-related glaucoma	current study

In summary, we confirm that *LTBP2* mutations are a recurrent cause of primary congenital megalocornea with zonular weakness and childhood lens-related secondary glaucoma. We suggest mutations in the gene do not typically cause primary congenital glaucoma; rather, that they cause this unique phenotype that resembles buphthalmos. Differentiation of congenital megalocornea with zonular weakness and childhood lens-related secondary glaucoma from primary congenital glaucoma is important, as lens removal surgery in indicated in the former while angle surgery is typically needed in the latter. Another feature of this unique phenotype is that early childhood spontaneous crystalline lens dislocation into the anterior chamber can occur, a phenomenon that was previously most associated with homocystinuria.
